# Late Corrective Arthrodesis in Nonplantigrade Diabetic Charcot Midfoot Disease Is Associated with High Complication and Reoperation Rates

**DOI:** 10.1155/2015/246792

**Published:** 2015-04-27

**Authors:** Anica Eschler, Georg Gradl, Annekatrin Wussow, Thomas Mittlmeier

**Affiliations:** ^1^Department of Trauma, Hand and Reconstructive Surgery, University of Rostock Medical Center, Schillingallee 35, 18057 Rostock, Germany; ^2^Department of Trauma, Orthopedic and Reconstructive Surgery, Munich Municipal Hospital Group, Harlaching Clinic, Sanatoriumsplatz 2, 81545 Munich, Germany

## Abstract

*Introduction*. Charcot arthropathy may lead to a loss of osteoligamentous foot architecture and consequently loss of the plantigrade alignment. In this series of patients a technique of internal corrective arthrodesis with maximum fixation strength was provided in order to lower complication rates. *Materials/Methods*. 21 feet with severe nonplantigrade diabetic Charcot deformity Eichenholtz stages II/III (Sanders/Frykberg II/III/IV) and reconstructive arthrodesis with medial and additional lateral column support were retrospectively enrolled. Follow-up averaged 4.0 years and included a clinical (AOFAS score/PSS), radiological, and complication analysis. *Results*. A mean of 2.4 complications/foot occurred, of which 1.5/foot had to be solved surgically. 76% of feet suffered from soft tissue complications; 43% suffered hardware-associated complications. Feet with only 2 out of 5 high risk criteria according to Pinzur showed significantly lower complication counts. Radiographs revealed a correct restoration of all foot axes postoperatively with superior fixation strength medially. *Conclusion*. Late corrective arthrodesis with medial and lateral column stabilization in the nonplantigrade stages of neuroosteoarthropathy can provide reasonable reconstruction of the foot alignment. Nonetheless, overall complication/reoperation rates were high. With separation into low/high risk criteria a helpful guide in treatment choice is provided. This trial is registered with German Clinical Trials Register (DRKS) under number DRKS00007537.

## 1. Introduction

Neuroosteoarthropathy (Charcot arthropathy) may lead to a loss of osteoligamentous foot architecture and consequently loss of the plantigrade foot alignment and midfoot or hindfoot instability, inducing subsequent soft-tissue complications such as skin breakdown, recurrent ulcerations, and infections [1, 2]. It is generally regarded that in the early stages of deformity, initial treatment should be nonoperative. However, patients at risk, presenting with signs of instability and progressive malalignment, show favourably low complication rates after reconstruction of foot alignment by reconstructive arthrodesis [1, 3, 4].

Reconstruction arthrodesis techniques vary from external fixation techniques using ring fixators to internal techniques with intra- and extramedullary implants such as plates, screws, or bolts or combinations of these [1, 4–7]. Postoperatively, prolonged healing periods due to diabetes-associated comorbidities such as peripheral artery disease can result in severe complications such as infections, nonunion or malunion, stress fractures, fixation failure, metal-induced soft-tissue irritations, implant breakage or loosening, and hence concomitant high reoperation rates [1, 6, 8–15]. Currently, general evidence-based treatment algorithms are lacking and the literature is inconsistent regarding both the ideal treatment type and timing of treatment.

In order to determine those at risk patients with nonplantigrade foot alignment for whom a favourable outcome is unlikely, Pinzur et al. [1, 9] in 2007 described a treatment algorithm based on his own experiences. Thus, high risk criteria such as a large bone deformity, a long-standing ulcer overlying infected bone, regional osteopenia, and obesity or immunocompromising illnesses, produced increased complication rates and therefore did not permit open reduction and internal fixation. Instead, percutaneous correction and fixation with an external ring fixator were recommended. Only in cases with low risk criteria was internal corrective arthrodesis recommended. Low risk criteria include the absence of open wounds, no history of deep infection, good bone quality, minimal diabetes-associated comorbidity, and the absence of morbid obesity [1, 9]. However, this algorithm was based on the experience of one single surgeon and low patient counts [1].

Since Pinzur's investigations, fixation devices have been further improved, and now locking plates, screws, and intramedullary placed rods and screws are utilized [5]. Despite this knowledge of advanced fixation techniques, postoperative complication rates are still high [5]. A recent investigation proved that fixation techniques using only medial column stabilization for midfoot deformity does not provide sufficient stability [16]. Consequently, additional lateral stabilization was postulated to provide increased and maximum stability [16].

In the present investigation, a series of neuroosteoarthropathic patients with corrective arthrodesis for late-stage Charcot midfoot neuroosteoarthropathy and medial and lateral column stabilization for maximum stability were enrolled and observed in a 4-year follow-up period. The aim of the study was to evaluate whether maximum fixation strength by medial and additional lateral column support could provide enough stability to lower the complication and amputation counts. Preoperative patient conditions were evaluated according to Pinzur's criteria [9].

## 2. Material and Methods

This retrospective review included 21 feet in 19 patients out of a consecutive series of 37 patients with severe Charcot neuroosteoarthropathy who underwent operative treatment by internal corrective arthrodesis, from November 2005 to March 2012. All patients were considered as patients at risk with either failed conservative treatment or nonamenability to successful conservative treatment due to imminent or persistent soft-tissue involvement in combination with radiological nonplantigrade foot alignment.

The indication criteria for corrective arthrodesis included the following: (1) a nonplantigrade foot alignment, (2) a high degree of instability of the medial and lateral midfoot region based on weight-bearing radiographs and radiological axes with positive talar-first metatarsal angle in anterior-posterior (AP) views, negative talar-first metatarsal angle in lateral views, negative calcaneal-fifth metatarsal angle in lateral views, and an increased dorsal midfoot displacement in lateral views [17], (3) clinically manifest or impending ulceration of soft tissues overlying bony deformity, and (4) failure of, or nonamenability to, successful conservative treatment. Inclusion criteria for the study were the above-mentioned indication criteria for corrective arthrodesis and intraoperative stabilization aiming for superior fixation strength by use of medial and additional lateral foot column stabilization adding to a minimum follow-up time of 1.5 years.

The mean age of the patients (14 male, 5 female) was 58.8 ± 8.5 months (range 29–76). All but 3 patients suffered from type 2 diabetes (*n* = 16, type 1 diabetes *n* = 3) with insulin dependency in 95% of patients (*n* = 18). Fifteen patients (79%) suffered from more than 3 secondary diagnoses with arterial hypertension (*n* = 15, 79%) in the first place, followed by obesity (*n* = 11, 59%, BMI 31.3 points), polyneuropathy (*n* = 9, 47%), and arterial occlusive disease (*n* = 7, 37%). Ulceration and imminent ulceration on locations of bony prominence affected 8 (38%) and 9 feet (43%), respectively, with 4 feet (19%) ulcer-free at time of surgery. Present ulceration came up to a medium extent of 2.1 cm^2^ (range 1–4) according to the subitem “E” of PEDIS classification [18] with a cumulative PEDIS count of 6.3 in the mean (range 3–11). According to Pinzur's criteria [1], 4 feet (19%) accounted for 2 out of 5 high risk criteria, 13 feet (62%) for 3 high risk criteria, and 4 feet (19%) for 4 positive high risk criteria (Table 1).

All but 3 feet (14%) were operated on during the consolidation phase Eichenholtz stage III. Applying the topographic classification of Sanders and Frykberg, a midfoot affection type II and/or III, corresponding to the Lisfranc and Chopart joints region, was diagnosed in all feet. Further, in 4 feet, an additional involvement of the subtalar and talocalcaneal joints according to Sanders/Frykberg type IV was visible in radiographs.

Preoperative assessment generally included color-coded duplex sonography in cases with doubt about the peripheral vascular status, aiming to reveal those patients with relevant macroangiopathy (angiographic dilatation and stenting was performed in 2 patients, resp.).

The synopsis of the radiological and clinical preoperative assessment indicated the region of corrective osteotomy, which was followed by stabilization using specific implants in order to achieve maximum stability. A medial utility incision exposed the medial column and talonavicular, navicular-cuneiform, and tarsometatarsal joints for either osteotomy, excision of the bony deformity, or denuding the joint surfaces from cartilage in preparation for fusion and reconstruction of a plantigrade foot position. Then, stabilization of the medial column was performed using extramedullary implants (*n* = 11, 52%; thereof *n* = 6, 29% angular stable plates) or intramedullary implants (*n* = 5, 24%) or a combination of both (*n* = 5, 24%). Afterwards, the stabilization of the lateral column was performed using extramedullary implants. Additional hindfoot arthrodesis was performed in 4 feet via compression screws (19%). In order to reconstruct the osseous foot, geometry resection of the necrotic midfoot bones was necessary in 2 feet (10%). In 14 feet (67%) the remaining osseous defects were filled with autologous iliac crest grafts. One foot (5%) required Achilles tendon lengthening as indicated by intraoperative evaluation of the tightness of the Achilles tendon complex. Intravenous prophylactic antibiotic (cefuroxime) was administered intraoperatively.

Postoperatively, a lower leg splint was applied which was replaced by a total contact cast from the time point when soft tissues showed consolidation, both with the recommendation of partial weight-bearing of 20 kg for 6 to 12 weeks postoperatively. Routine follow-up, for example, for cast changes and radiographs, took place 2 weeks after discharge from hospital and then at monthly intervals up to bony consolidation, followed by follow-up visits once a year. Mean follow-up averaged 4.0 years (range 1.8–7.4). Three patients deceased of causes independent of foot surgery and therefore only available for radiological and complication analyses. In all, 21 feet did not meet the inclusion criteria and were therefore excluded from follow-up; in particular type of stabilization was causal; in 15 feet solely medial column stabilization and in 6 feet solely hindfoot stabilization were performed.

Follow-up included detailed failure analyses for the perioperative and postoperative time period, focusing on complication and reoperation rates. Therefore, early (i.e., 30 days after surgery), intermediate (30 days–5 months after surgery), and late complications (from the 6th month following surgery) were recorded, as well as the need for further surgery. Complication analyses focused on soft-tissue complications, implant-associated complications, nonunion (stable/nonstable), and amputation. Nonunion was defined as a delay in healing evident for at least 6 months postoperatively. Impaired wound healing was defined as wound healing deviating from normal wound healing, for example, prolonged secretion or wound dehiscence leading to a length in hospitalization or further intervention. Impaired wound healing can be caused by infection but not necessarily; infection was defined with evident bacterial/fungal contamination. Superficial wound infections occur in cutaneous/subcutaneous tissue surrounding the surgical incision. Deep wound infections extend into the below and besides laying muscle tissue/fasciae and have potential to further develop to osteomyelitis. Cases of minor and major revision surgery were considered separately. Each patient completed the AOFAS midfoot scale [19] as well as the patient satisfaction survey (PSS) as described by Grant et al. [15]. For the latter 4 categorical questions were asked:* “(1) How is the foot? (2) Can you walk? (3) Do you experience pain? (4) Is the foot stable?”*. Furthermore, consultation frequency, patient mobilization, and detailed radiological follow-up were registered. For the latter the talar-first metatarsal angle in AP views and lateral talar-first metatarsal angle, calcaneal-fifth metatarsal angle, and dorsal midfoot displacement were evaluated in pre-, post-, and follow-up weight-bearing radiographs to outline the resulting correction of deformity and maintenance of correction [17]. Whilst the mean values are given in absolute numbers representing the change of angulation for the AP talar-first metatarsal angle, the range is given with algebraic signs; thus negative values correspond to abduction deformity and positive values correspond to adduction deformity of the forefoot.

*Statistical Analysis*. Results were given as mean ± SEM (range). After proving the assumption of normality (Kolmogorov-Smirnov test), dependent *t*-test analyses or Mann-Whitney *U* test (nonnormal distribution) was performed to analyze the differences in radiographic parameters. Significance was defined at *p* < 0.05. Statistical testing was performed using IBM SPSS Statistics version 20.0 software (Armonk, New York, USA).

## 3. Results

Mean time of hospitalization following the initial operation was 33.9 ± 5.3 days (range 8–86). Mean time of hospitalization during the whole follow-up period including times for complication management was 59.1 ± 8.7 days (range 8–156). Mean postoperative casting period was 2.7 ± 0.4 months (range 1–7).

At final follow-up, 14% of patients (*n* = 3) were fully mobile without walking aids of whom 9 patients' feet (43%) used certain orthopaedic shoes. Four patients (19%) wore a prosthesis. Only 1 patient (5%) used a rollator and 1 patient (5%) a cane. Here and as follows the term “patient” is used as a synonym for case/foot.

### 3.1. Complications

A total of 13 out of 21 patients (62%) suffered from early complications; 12 patients (57%) suffered from intermediate complications and 10 patients (48%) from late complications. Only 2 patients were complication-free within the 4-year follow-up period (Table 1).

### 3.2. Soft-Tissue Complications

Sixteen patients (76%) suffered from soft-tissue complications during the follow-up period. In detail, superficial wound infections were observed in 6 patients with 8 feet (38%), followed by 6 patients with 8 feet (38%) with impaired wound healing, and 7 patients with 7 feet (33%) with recurrent ulceration. In 3 patients with 3 feet (14%), deep wound infection escalated to osteomyelitis. Of all soft-tissue complications, 38% (*n* = 14) occurred during the late follow-up phase, 31% (*n* = 11) within the early phase, and 31% (*n* = 11) within the intermediate phase (Table 2).

### 3.3. Implant-Associated Complications and Nonunion

Nine patients with 9 feet (43%) suffered from hardware-associated complications with 2 feet (10%) of hardware loosening and 7 feet (33%) of hardware breakage. In 1 foot (5%), nonunion of the arthrodesis region was observed. Furthermore, 50% of all implant-associated complications occurred within the intermediate phase; however, 20% had already been observed within the early postoperative phase (Table 2).

### 3.4. Amputation

Amputation was performed in 5 patients with 5 feet (24%), with a need for lower leg amputation in 4 feet and for forefoot amputation (Chopart) in 1 foot. Therefore, the annual complication rate was 6%. Amputation was performed in a mean of 1.0 ± 0.2 years (range 0–3) after initial surgery. All amputated patients showed 3 out of 5 positive high risk criteria as stated by Pinzur [9].

### 3.5. Complication Management

In the synopsis, each patient suffered from a mean of 2.4 ± 0.3 complications (range 0–6, *n* = 53) of which 1.5 ± 0.3 (range 0–4, 66%) had to be solved surgically. Only 5 patients (24%) did not need further surgery, of which 2 patients (10%) did not need any further therapy at all and 3 patients (14%) successfully recovered with nonsurgical complication management. Feet with only 2 out of 5 positive high risk criteria according to Pinzur [9] showed lower complication rates, with a mean of 1.3 complications (range 0–2, *n* = 5). Patients with 3 to 5 positive high risk criteria according to Pinzur [9] suffered from 2.8 ± 0.3 complications (range 0–6, *n* = 48) in mean (*p* = 0.09).

In 7 patients (33%), only minor revision surgery such as soft-tissue debridement or jet lavage was necessary. In 8 patients (38%), major surgery such as rearthrodesis or amputation was performed, of which 7 patients (33%) underwent minor revision surgery during follow-up.

Concerning the time point of complication management, 57% of early complications, 69% of intermediate, and 68% of late complications needed surgical complication management. In all, 41% of all complications were successfully treated with conservative management.

### 3.6. Patient Satisfaction

The mean AOFAS midfoot score accounted for 60 ± 2.5 points (range 44–76) at final follow-up revealing a good overall patient satisfaction. Considering the subcategories, “pain” accounted for 29 ± 1.9 points (range 10–40), “function” for 23 ± 2.1 points (range 12–42), and “alignment” for 7 ± 1.1 points (range 0–15). Outcome for the patient satisfaction survey (PSS) was in line with those results: The question* “How is the foot?”* was reported to be “better” in 8 (50%) out of 16 available patient records for follow-up; 6 (38%) patients reported it is the “same.” “*Can you walk”* was answered by all patients except one (amputated) with “yes.”* “Do you experience pain?”* was reported by 11 patients (69%) with “no” and 9 patients (56%) had a stable feeling in weight bearing.

### 3.7. Radiological Results

The mean talar-first metatarsal angle in AP views, as an indicator for abduction/adduction midfoot deformity, improved from 7.5 ± 0.8° (range −18–+12) preoperatively to 4.3 ± 1.1° (range −7–+23) postoperatively and did not show a relevant collapse with a final 4.3 ± 0.7° (range −8–+10) angulation at follow-up.

The mean lateral talar-first metatarsal angle showed significant improvements from −17.3 ± 0.8° (range −29–−7) preoperatively to −0.3 ± 1.5° (range −12–+13) postoperatively (*p* < 0.01). At final follow-up, a loss of angulation to −9.1 ± 2.2° (range −28–+10) was found; hence this loss of reduction was significant (*p* < 0.01). Ideally, the intraoperative change of the lateral talar-first metatarsal angle should be zero, corresponding to a change from valgus deformity to the neutral axis and therefore anatomic erection of the foot arch.

The same rule, a high positive value postoperative correction, applies for the calcaneal-fifth metatarsal angle which displays the lateral foot arch in the lateral view. The calcaneal-fifth metatarsal angle improved from 7.2 ± 1.9° (range −8–+19) preoperatively to 16.0 ± 1.7° postoperatively (range 4–28, *p* < 0.01). For final follow-up, it decreased to 2.8 ± 1.4 (range −11–+10), corresponding to a recollapsed lateral foot arch (*p* < 0.01).

Since standard angles on lateral radiographs tend to underestimate the grade of deformity with midfoot joint displacement [17], the dorsal midfoot displacement (lateral view X-ray) displaying the vertical distance at the level of dislocation within the talar midline axis and the first metatarsal axis was measured. Dorsal midfoot displacement showed significant postoperative improvements from 18.4 ± 2.2 mm (range 2–35) to 5.9 ± 0.9 mm (range 2–13) and finally a loss of reduction to 10.7 ± 1.5 mm (range 4–25) at follow-up (*p* < 0.01).

## 4. Discussion

The treatment of diabetic neuroosteoarthropathy is one of the most challenging problems facing the orthopaedic community and still a matter of controversy. In particular, in the case of nonplantigrade alignment of the midfoot and hindfoot, an especially high rate of skin breakdown and ulcers at the site of bony deformities are observed [1, 2]. Pinzur [1, 20] and other authors [21, 22] have proposed the following current main goals in the treatment of Charcot feet: a long-time infection-free and ulcer-free foot with the ability to use commercially available depth-inlay shoes and custom-accommodative foot orthoses maintaining a long-term walking independence.

Reconstructive surgery has been suggested by several authors as a valuable treatment option for severe deformity [4, 23–25]. Until now, there has been no relevant evidence-based literature, but clinical reports indicate that stability can be restored with precise surgical technique, appropriate perioperative education, and postoperative therapy, assuming adequate patient compliance [5, 24]. Nevertheless, complications such as infections, reulcerations, nonunion/malunion, fixation failure, implant breakage/loosening, and recurrence of deformity are frequently reported in the literature and indicate an overall complication rate of >30% with surgical interventions [1, 6, 8–15]. Baravarian and Van Gils [24] included 14 clinical series in their review of Charcot foot arthrodesis and found that 25% of the reported procedures including application of an external ring fixator and plate or screw fixation were subject to at least one complication. Lowery et al. [5] recently found in their literature review of 95 level IV and V studies a 22.4% nonunion rate after reconstructive surgeries. Eventually, all kinds of fixation methods are likely to cause complications; intramedullary positioned implants as rods or screws are accompanied by implant loosening, bolt migration, and breakage [26–28] leading to high complication rates of up to 30% [1, 8, 24]. External ring fixators avoid internal positioning of implants on the one hand, but on the other hand if positioned in a stand-alone technique they are associated with complicating pin breakages and pin infections, compounding a difficult intraoperative reconstruction [9–15]. Again, high rates of infectious complications adding to a high degree of patient discomfort are the result [1, 6, 29]. Therefore, Sammarco [30] in 2009 introduced the term “superconstruct” reflecting the need for a more effective fixation technique implementing the following 4 characteristics: (1) fusion extension beyond the conflict region to improve fixation, (2) deformity resection with consecutive bony shortening for decreased tissue tension, (3) strongest implant still tolerated by the surrounding tissues, and (4) device positioning with optimal biomechanical effects.

By stabilization of the medial and lateral column in midfoot instability and with respect to all 4 above-mentioned criteria, we aimed to reach maximum stability and have retrospectively examined 21 patients in a 4-year follow-up period. Despite the assumption of maximum stability, high complication and amputation rates were observed. Soft-tissue complications turned out to affect all postoperative phases similarly and accounted for 76% of complications of feet with 38% superficial wound infections in the first place. According to several authors this appears to be attributable to the endangered diabetic patient collective [31] and the results are in line with other groups reporting a range of 7–26% risk for wound infections in their 2.5-year follow-up [15, 32]. Recently a prospective study revealed that neuropathy as well as poorly controlled diabetes is associated with increased surgical site infection rates: patients with uncontrolled diabetes had a 7.25-fold increased infection risk compared with nondiabetic nonneuropathic patients and a 3.72-fold increased risk compared with patients with uncomplicated diabetes [33]. Despite adequate management of those superficial soft-tissue complications, they escalated in 14% to deep infections and osteomyelitis explaining the unpleasant length of hospital stay of 34 days in mean. Osteomyelitis rates for reconstructive surgeries in Charcot feet were reported with 12–16% [15, 26]. One might argue if a postoperative casting longer than 2.7 months might have reduced complication rates; unfortunately there are no studies in the literature reporting on the effect of different casting modalities and duration. However, complications are not a domain for surgical treatment in Charcot feet. With conservative treatment, 30% of patients experience recurrence of soft-tissue problems just within the period of change from casts to footwear [34]. Moreover, osteomyelitis rates of 33% have been reported after conservatively treated diabetic foot infections [35]. Those high rates naturally argue in favour of reconstructive surgery in patients at risk.

In this study, the second most common complication following soft-tissue complications was implant-associated problems. These most often occurred within the intermediate postoperative phase (1–5 months after surgery) and accounted for 43% of complications during the whole follow-up period. Anyhow, 20% occurred already in the early phase and were reducible to unfavourable diabetic patient conditions as obesity and unreliability to partial weight bearing. This is in line with other studies, for example, Myers et al. [36] who observed an increased risk for postoperative complications after foot and/or ankle arthrodesis in diabetic patients, when compared to nondiabetic patients and Sammarco et al. [17] who observed similar rates with 32% implant breakage in their 4.3-year follow-up after midtarsal arthrodesis. A recent review [5] including 95 level IV and V studies revealed similar results and ulcer-free feet in most cases but with 22.4% nonunion rates. In any case, nowadays there is consensus that even with incomplete union a sufficiently stable and an ulcer-free plantigrade foot may be achieved [5]. This finding is endorsed by our patient satisfaction evaluation revealing overall good satisfaction for final follow-up in the AOFAS midfoot and PSS scores. Anyhow, both scores were only obtained for final follow-up outlining a weakness of the study.

We observed 5 patients (24%) with the need for amputation leading to an annual amputation rate of 6%. These high rates are relativized by the recent finding of Wukich and Pearson [37] who actually reported an improvement of neuropathic patients' self-reported quality of life following transtibial amputation. Our amputation rates are in line with Saltzman et al. [23] who reported, for a cohort of 115 patients and a 3.8-year follow-up, an annual amputation risk of 3% without and an amputation risk of 28% with ulceration present initially, as well as a 23% risk of requiring more than 18 months casting and a high risk of about 49% for recurrent ulceration. In our patient collective, 33% of patients suffered from recurrent ulcerations. Illgner et al. [7] reported on 205 patients in a follow-up period of 21 months who were treated with an external ring fixator and observed a reulceration rate of 25%.

It seems that even a high degree of stability with internal reconstructive arthrodesis techniques using medial and lateral column support with intra- and extramedullary implants cannot provide improved results in terms of complication and amputation rates when compared to techniques as previously described. All our patients had at least 2, and in the mean 3, positive high risk criteria according to Pinzur [9]. Pinzur [9] in 2007 stated that patients with substantial bone deformity, a long-standing ulcer overlying infected bone, regional osteopenia, and obesity or immunocompromising illnesses are not qualified for open reduction and internal fixation (=5 high risk criteria). Only in case of present low risk criteria, such as absence of open wounds, no history of deep infection, good bone quality, minimal diabetes-associated comorbidity, and no morbid obesity, internal reconstructive arthrodesis was recommended [9]. In any case, there was no detailed algorithm provided and therefore it remained unclear how many positive high or low risk criteria qualify for corrective arthrodesis. Our patient collective revealed lower complication counts (1.3 complications/patient) in feet with only 2 out of 5 positive high risk criteria when compared to the mean complication count of 2.4 complications, thus leading to a rate of 2.8/patient for those patients with more than 2 positive high risk criteria. All patients who had to undergo amputation during the further clinical course showed at least 3 positive high risk criteria. Therefore, from present viewpoint, patients with 1 to 2 positive high risk criteria may be suitable for internal reconstructive surgery, although larger clinical trials are needed to verify this finding.

In terms of radiological evaluation, the goal of reconstruction was to correct the nonplantigrade foot position and to establish a neutral anterior-posterior-talar-first metatarsal angle, positive lateral talar-first metatarsal angle and calcaneal-fifth-metatarsal angle, and dorsal midfoot displacement correction to normal values [38]. Intraoperative improvement of the lateral talar-first metatarsal angle succeeded significantly, but a certain loss of reduction was seen in final follow-up, however still with improved angulation when compared to preoperative values. With medial and lateral column support for maximum stabilization according to Sammarco's criteria [17] enough fixation strength is provided to hold the reconstruction of the medial foot column. Nevertheless, this does not seem to provide enough fixation strength for the lateral column: angulation in lateral talar-first metatarsal angle was significantly improved, but a severe recollapse had to be observed in our collective at final follow-up, even with fixation using angular stable plates in 29% of patients. At least, the final radiographic result was improved when compared to preoperative values. The same applies for the dorsal midfoot displacement which is well in line with other studies [17, 26]. The calcaneal fifth-metatarsal angle showed significant improvements postoperatively, while a severe recollapse had to be observed later on. Wiewiorski et al. [26] analogously experienced the worst recollapse for the lateral talar-first metatarsal and calcaneal-fifth-metatarsal angles. Wukich et al. [38] recently revealed similar results for lateral column involvement proven in changes of the lateral calcaneal-fifth-metatarsal angle, calcaneal pitch, and cuboid height and found an increased risk for ulceration when compared to solely medial column involvement.

## 5. Conclusion

Late corrective arthrodesis with medial and lateral column stabilization in nonplantigrade Charcot midfoot neuroosteoarthropathy can provide reasonable reconstruction of the foot alignment with adequate stability. During a 4-year follow-up period, correction maintenance of the medial column was superior; however, the lateral column showed less stability with a tendency to recollapse. Despite the good radiological results, complication and reoperation rates as well as amputation rates did not show superior results compared to concepts with inferior stability such as solely medial or lateral column stabilization. The Pinzur [1, 9] criteria separated into low and high risk criteria may give helpful clinical orientation as to the choice of treatment: feet with more than 3 positive Pinzur high risk criteria showed inferior outcome results than did those with 2 positive criteria. This might provide an argument against selecting internal fixation in high risk patients.

## Figures and Tables

**Table 1:** Patients' characteristics and classification according to Pinzur's high risk criteria and complication counts [1, 9].

Number	Age	Gender	Diab. type	Complications	Classification		High risk criteria					
					Sanders/Frykberg	Eichenholtz	Immunocompromising illnesses	Large bone deformity	Longstanding ulcer	Regional osteopenia	Obesity	Σ
1^∗^	65	M	II	**4**	III	2	—	x	—	x	x	**3**
2^∗^	64	M	II	**2**	III	3	—	x	—^∗∗^	x	—	**2**
3^∗^	66	W	II	**4**	II/III	3	—	x	x	x	x	**4**
4^∗^	69	W	II	**2**	II/III	3	—	x	x	x	—	**3**
5	56	M	II	**0**	II/III	3	—	x	—^∗∗^	x	x	**3**
6	76	W	II	**3**	III	3	—	x	—^∗∗^	x	x	**3**
7	48	M	I	**2**	II/III	3	—	x	x	x	—	**3**
8	55	M	II	**1**	III/IV	3	—	x	—^∗∗^	x	x	**3**
9	46	M	II	**3**	II/III	3	—	x	—^∗∗^	x	x	**3**
10	59	M	II	**3**	II/III	3	—	x	—^∗∗^	x	x	**3**
11	61	M	II	**0**	II/III	2	—	x	—	x	x	**3**
12	49	M	II	**1**	II/III	3	—	x	—^∗∗^	x	x	**3**
13	62	M	II	**4**	III/IV	3	x	x	—	x	—	**3**
14	40	W	I	**1**	II/III	3	—	x	—^∗∗^	x	—	**2**
15	53	M	II	**1**	III	2	—	x	—^∗∗^	x	—	**2**
16	69	W	II	**6**	II/III	3	—	x	x	x	x	**4**
17	66	M	II	**3**	II–IV	3	x	x	—^∗∗^	x	—	**3**
18	59	M	I	**4**	II–IV	3	—	x	x	x	—	**3**
19	68	W	II	**1**	II/III	3	—	x	—	x	—	**2**
20	46	M	II	**3**	II/III	3	—	x	—^∗∗^	x	x	**3**
21	58	M	II	**2**	III	3	—	x	x	x	—	**3**

^∗^Patients with inclusion of both feet; ^∗∗^imminent ulceration.

**Table 2:** Complications in study patients.

Complications	All (feet)		Early (counts)	Intermediate (counts)	Late (counts)
	*n*	%	*n*	*n*	*n*
Soft-tissue complications *n* = 16 feet					
Impaired wound healing	**8**	**38**	6	2	2
Superficial wound infect.	**8**	**38**	4	6	2
Deep wound infection/osteomyelitis	**3**	**14**	—	1	2
Reulceration	**7**	**33**	—	2	8
Hematoma	**1**	**5**	1	—	—

Implant-associated complications and nonunion *n* = 9 feet					
Implant loosening	**2**	**10**	—	2	—
Implant breakage	**7**	**33**	2	3	3
Nonunion	**1**	**5**	—	—	1
Other (pain, discomfort)	**2**	**10**	1	—	1

## References

[1] Pinzur M. S., Sostak J. (2007). Surgical stabilization of nonplantigrade Charcot arthropathy of the midfoot. *American Journal of Orthopedics*.

[2] Robinson A. H. N., Pasapula C., Brodsky J. W. (2009). Surgical aspects of the diabetic foot. *The Journal of Bone & Joint Surgery—British Volume*.

[3] Alpert S. W., Koval K. J., Zuckerman J. D. (1996). Neuropathic arthropathy: review of current knowledge. *The Journal of the American Academy of Orthopaedic Surgeons*.

[4] Mittlmeier T., Klaue K., Haar P., Beck M. (2010). Should one consider primary surgical reconstruction in Charcot arthropathy of the feet?. *Clinical Orthopaedics and Related Research*.

[5] Lowery N. J., Woods J. B., Armstrong D. G., Wukich D. K. (2012). Surgical management of charcot neuroarthropathy of the foot and ankle: a systematic review. *Foot and Ankle International*.

[6] Koller A., Hafkemeyer U., Fiedler R., Wetz H. H. (2004). Reconstructive foot surgery in cases of diabetic-neuropathic osteoarthropathy. *Orthopade*.

[7] Illgner U., Podella M., Rümmler M., Wühr J., Büsch H. G., Wetz H. H. (2009). Reconstructive surgery for Charcot foot: long-term 5-year outcome. *Orthopade*.

[8] Pakarinen T.-K., Laine H.-J., Honkonen S. E., Peltonen J., Oksala H., Lahtela J. (2002). Charcot arthropathy of the diabetic foot. Current concepts and review of 36 cases. *Scandinavian Journal of Surgery*.

[9] Pinzur M. S. (2007). Neutral ring fixation for high-risk nonplantigrade charcot midfoot deformity. *Foot and Ankle International*.

[10] Farber D. C., Juliano P. J., Cavanagh P. R., Ulbrecht J., Caputo G. (2002). Single stage correction with external fixation of the ulcerated foot in individuals with Charcot neuroarthropathy. *Foot and Ankle International*.

[11] Sayner L. R., Rosenblum B. I. (2005). External fixation for Charcot foot reconstruction. *Current Surgery*.

[12] Marks R. M., Parks B. G., Schon L. C. (1998). Midfoot fusion technique for neuroarthropathic feet: biomechanical analysis and rationale. *Foot and Ankle International*.

[13] Dalla Paola L., Ceccacci T., Ninkovic S., Sorgentone S., Marinescu M. G. (2009). Limb salvage in charcot foot and ankle osteomyelitis: combined use single stage/double stage of arthrodesis and external fixation. *Foot and Ankle International*.

[14] Cooper P. S. (2002). Application of external fixators for management of Charcot deformities of the foot and ankle. *Foot and Ankle Clinics*.

[15] Grant W. P., Garcia-Lavin S. E., Sabo R. T., Tam H. S., Jerlin E. (2009). A retrospective analysis of 50 consecutive Charcot diabetic salvage reconstructions. *Journal of Foot and Ankle Surgery*.

[16] Eschler A., Wussow A., Ulmar B., Mittlmeier T., Gradl G. (2014). Intramedullary medial column support with the Midfoot Fusion Bolt (MFB) is not sufficient for osseous healing of arthrodesis in neuroosteoarthropathic feet. *Injury*.

[17] Sammarco V. J., Sammarco G. J., Walker E. W., Guiao R. P. (2009). Midtarsal arthrodesis in the treatment of charcot midfoot arthropathy. *The Journal of Bone & Joint Surgery—American Volume*.

[18] Lipsky B. A., Berendt A. R., Cornia P. B. (2012). 2012 infectious diseases society of America clinical practice guideline for the diagnosis and treatment of diabetic foot infections. *Clinical Infectious Diseases*.

[19] Kitaoka H. B., Alexander I. J., Adelaar R. S., Nunley J. A., Myerson M. S., Sanders M. (1994). Clinical rating systems for the ankle-hindfoot, midfoot, hallux, and lesser toes. *Foot and Ankle International*.

[20] Pinzur M. (2004). Surgical versus accommodative treatment for charcot arthropathy of the midfoot. *Foot and Ankle International*.

[21] Lamm B. M., Gottlieb H. D., Paley D. (2010). A two-stage percutaneous approach to charcot diabetic foot reconstruction. *Journal of Foot and Ankle Surgery*.

[22] Grant W. P., Garcia-Lavin S., Sabo R. (2011). Beaming the columns for Charcot diabetic foot reconstruction: a retrospective analysis. *Journal of Foot and Ankle Surgery*.

[23] Saltzman C. L., Hagy M. L., Zimmerman B., Estin M., Cooper R. (2005). How effective is intensive nonoperative initial treatment of patients with diabetes and Charcot arthropathy of the feet?. *Clinical Orthopaedics and Related Research*.

[24] Baravarian B., Van Gils C. C. (2004). Arthrodesis of the Charcot foot and ankle. *Clinics in Podiatric Medicine and Surgery*.

[25] Garapati R., Weinfeld S. B. (2004). Complex reconstruction of the diabetic foot and ankle. *American Journal of Surgery*.

[26] Wiewiorski M., Yasui T., Miska M., Frigg A., Valderrabano V. (2013). Solid bolt fixation of the medial column in charcot midfoot arthropathy. *Journal of Foot and Ankle Surgery*.

[27] Popelka S., Hromádka R., Barták V., Sosna A. (2012). Bilateral arthrodesis of the medial foot joints in a patient with rheumatoid arthritis. *Acta Chirurgiae Orthopaedicae et Traumatologiae Cechoslovaca*.

[28] Wiewiorski M., Valderrabano V. (2012). Intramedullary fixation of the medial column of the foot with a solid bolt in Charcot midfootarthropathy: a case report. *Journal of Foot and Ankle Surgery*.

[29] Wukich D. K., Sung W. (2009). Charcot arthropathy of the foot and ankle: modern concepts and management review. *Journal of Diabetes and Its Complications*.

[30] Sammarco V. J. (2009). Superconstructs in the treatment of charcot foot deformity: plantar plating, locked plating, and axial screw fixation. *Foot and Ankle Clinics*.

[31] Sohn M.-W., Stuck R. M., Pinzur M., Lee T. A., Budiman-Mak E. (2010). Lower-extremity amputation risk after Charcot arthropathy and diabetic foot ulcer. *Diabetes Care*.

[32] Bevilacqua N. J., Rogers L. C., Armstrong D. G. (2008). Diabetic foot surgery: classifying patients to predict complications. *Diabetes/Metabolism Research and Reviews*.

[33] Wukich D. K., Crim B. E., Frykberg R. G., Rosario B. L. (2014). Neuropathy and poorly controlled diabetes increase the rate of surgical site infection after foot and ankle surgery. *Journal of Bone and Joint Surgery—American Volume*.

[34] Bates M., Petrova L., Edmonds M. E. (2006). How long does it take to progress from cast to shoes in the management of Charcot osteoarthropathy?. *Diabetic Medicine*.

[35] Kaynak G., Birsel O., Fatih Güven M., Öğüt T. (2013). An overview of the Charcot foot pathophysiology. *Diabetic Foot and Ankle*.

[36] Myers T. G., Lowery N. J., Frykberg R. G., Wukich D. K. (2012). Ankle and hindfoot fusions: comparison of outcomes in patients with and without diabetes. *Foot and Ankle International*.

[37] Wukich D. K., Pearson K. T. (2013). Self-reported outcomes of trans-tibial amputations for non-reconstructable Charcot neuroarthropathy in patients with diabetes: a preliminary report. *Diabetic Medicine*.

[38] Wukich D. K., Raspovic K. M., Hobizal K. B., Rosario B. (2014). Radiographic Analysis of Diabetic Midfoot Charcot Neuroarthropathy With and Without Midfoot Ulceration. *Foot & Ankle International*.

